# Code of Medical Ethics, Adopted by the American Medical Association

**Published:** 1867-05

**Authors:** 


Code of Medical Ethics, adopted by the American Medical Association. New York: WM. WOOD & Co. 1867.

The publishers have furnished the profession with the *Code*

*of Ethics* in the form of a small duodecimo volume, neatly printed and bound. A copy of it should be purchased and carefully read by every practising physician and surgeon in the United States. For sale by WM. WOOD & Co., 61 Walker St., New York City.

